# Supplementary material for: Utilizing Interactive Surfaces to Enhance Learning, Collaboration and Engagement: Insights from Learners’ Gaze and Speech
Source: Sensors (Basel). 2020 Mar 31;20(7):1964. doi: 10.3390/s20071964 (PMC7180823; doi:10.3390/s20071964)
Supplement: Supplementary file 1 [file sensors-20-01964-s001.pdf]

sensors

MDPI

Supplementary Material

Article

Utilizing Interactive Surfaces to Enhance Learning, Collaboration and Engagement: Insights from Learners' Gaze and Speech

Kshitij Sharma *, Ioannis Leftheriotis and Michail Giannakos

Department of Computer Science, Norwegian University of Science and Technology, Trondheim 7491, Norway; midmandy@gmail.com (I.L.); michailg@ntnu.no (M.G.)
* Correspondence: kshitij.sharma@ntnu.no

Pre-test

A. Gender:
B. Age:
C. Previous experience with technology

1	2	3	4	5	6	7
Not at all						Very Much

D. Previous experience with touch screens (e.g., big screens, ipads, mobile devices)

1	2	3	4	5	6	7
Not at all						Very Much

E. Previous experience with big touch screens (bigger than a laptop size)

1	2	3	4	5	6	7
Not at all						Very Much

1. Which image shows the correct placement of the parietal and temporal lobes?

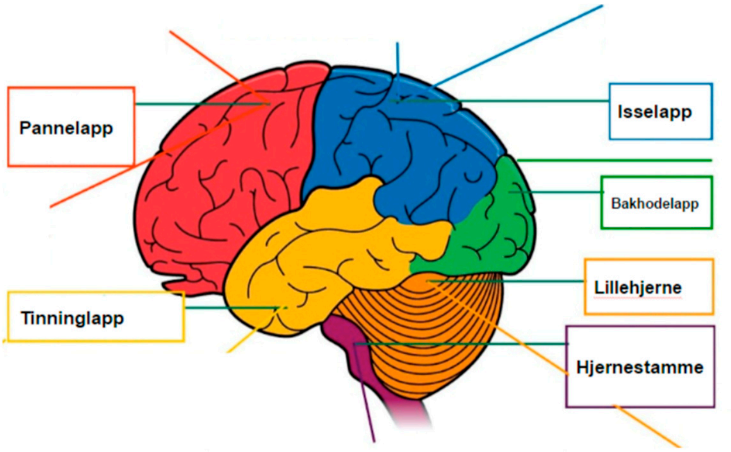

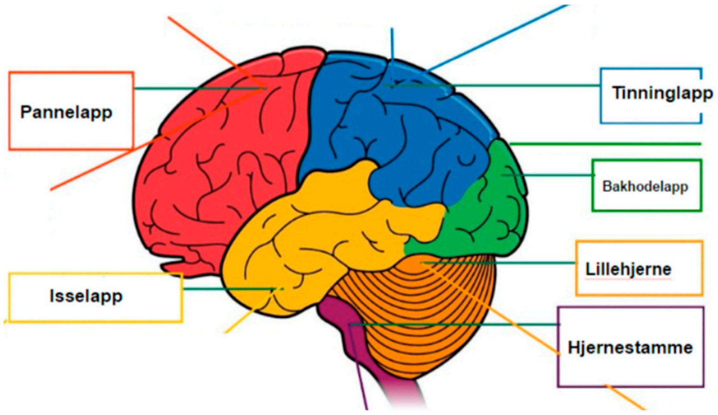

2. More boys than girls have dyslexia

False, longitudinal research shows as many girls are affected as boys

True, this is linked with boys neurologically maturing later

3. Dyslexia affects __ % of the population and often occurs with ____

4. Which part of the brain is most closely associated with ADHD?

left-sided prefrontal cortex

right-sided prefrontal cortex

5. What is the cause of ADHD?

In a majority of cases, this is unfortunately unknown

The vast majority of cases have a causal link to population density

6. Which image shows the part of the brain affected by temporal lobe epilepsy?

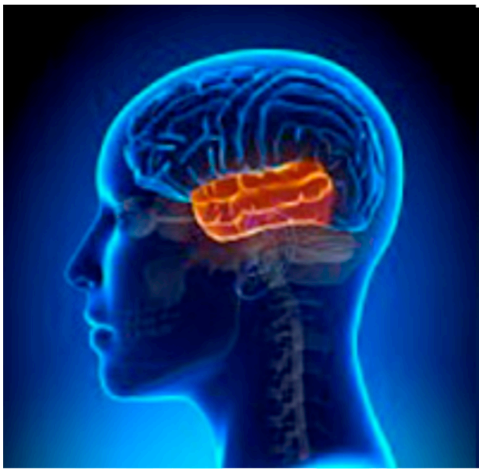

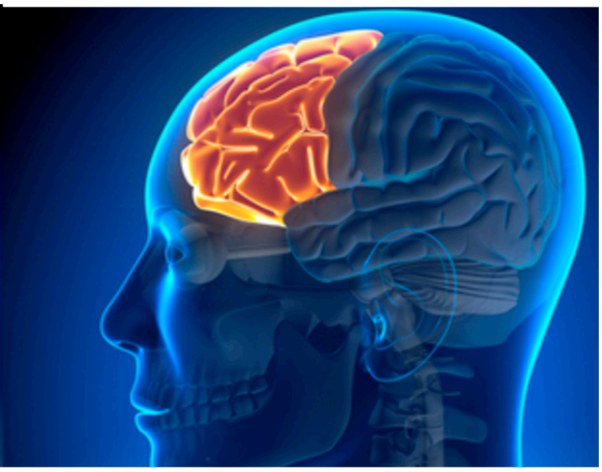

7. What is the amygdala responsible for?

Decision making

Long-term memory

8. What the thalamus responsible for?

Short term memory

Sleep

9. Which image shows the thalamus?

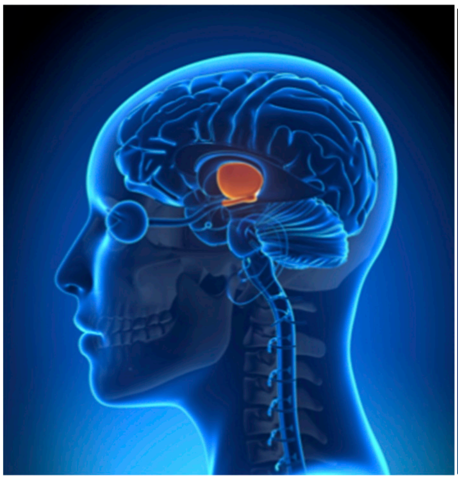

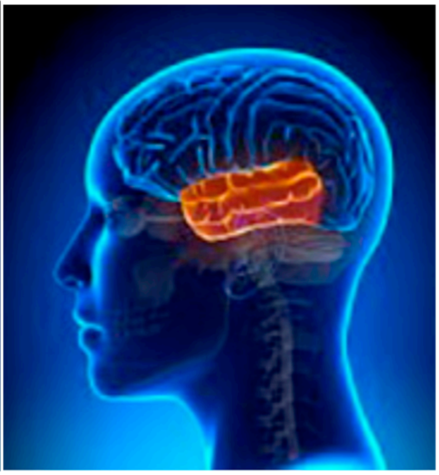

10. Which part of the brain is responsible for language and attention?

Cerebellum

Hippocampus

First post-test

1. Which gland produces insulin?

Pancreas

Pituitary

2. What are the common symptoms of dyslexia?

Trouble reading

Insomnia

3. Which brain functions are affected by ADHD?

The left-sided prefrontal cortex.

The right-sided prefrontal cortex.

4. Which image shows the part of the brain associated with ADHD?

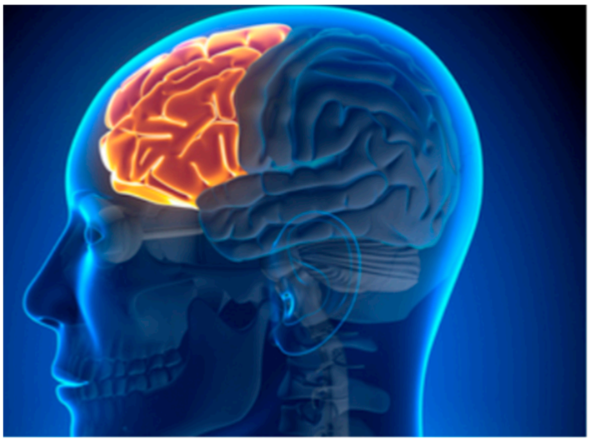
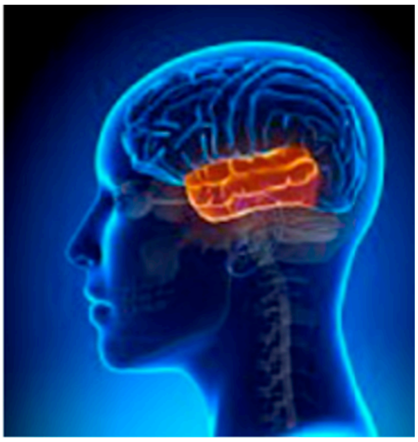

5. What is the most common cause of temporal lobe epilepsy?

Traumatic brain injury

Headache

6. What treatments are effective for sufferers of temporal lobe epilepsy?

Antiepileptic drugs and surgical interventions

No treatment

7. Which region of the brain stores long term memory?

Hippocampus

Thalamus

8. What is the function of the hypothalamus

Body temperature

Emotions

9. Which part of the brain controls body temperature?

Hypothalamus

Amygdala

10. The cerebellum is responsible for ____

Language and attention

Short time memory

Second post-test

1. What is the name of the hormone the testes produce?

Testosterone

Insuline

2. Which image shows the part of the brain associated with dyslexia?

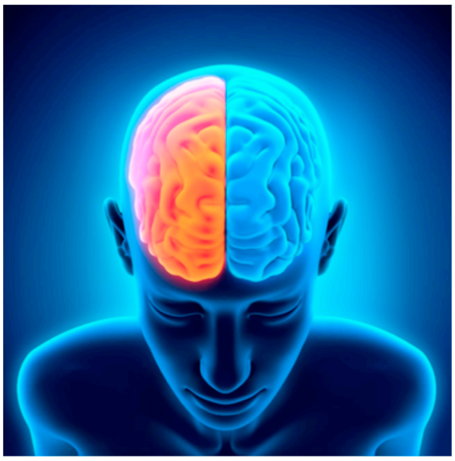

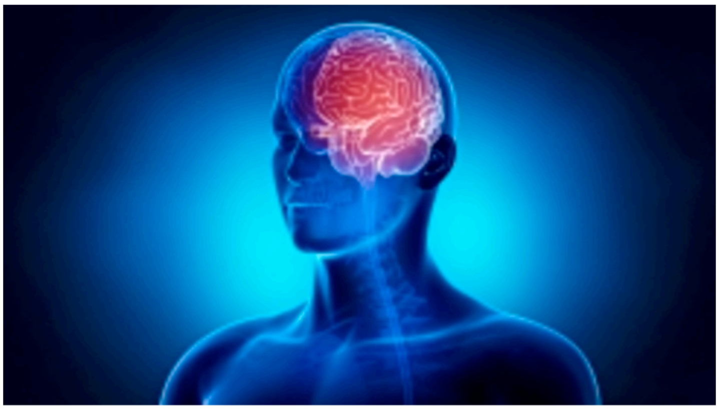

3. What is the most common symptom of ADHD?

Problems paying attention

Abnormal aging

4. The temporal lobe is associated with temporal lobe epilepsy, which image points to the temporal lobe?

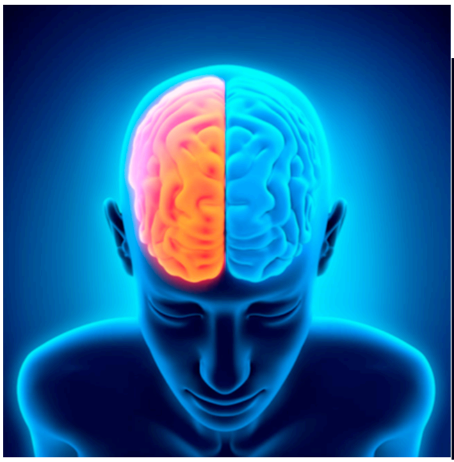

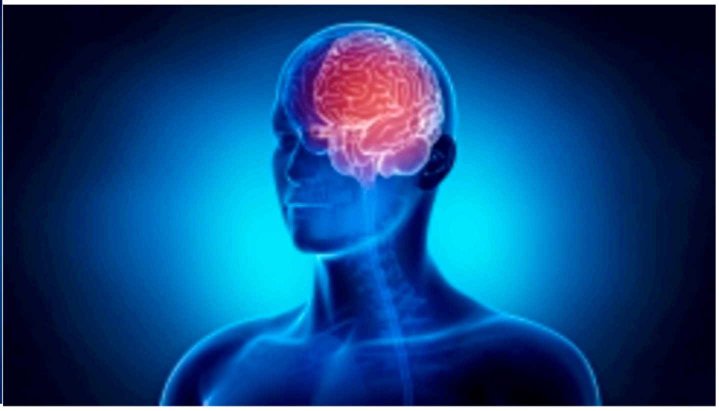

5. What is the most common symptom of temporal lobe epilepsy?

Lack of attention

Simple partial seizures

6. Which part of the brain is responsible for long-term memory?

Hypothalamus

Hippocampus

7. Which part of the brain regulates body temperature and hunger control?

Hypothalamus

Hippocampus

8. What is the function of the cerebellum?

Language and attention

Sleep and sensory interpretation

9. What is the function of the thalamus?

Language and attention

Sleep and sensory interpretation

10. What is the most common name for neurons?

Nerve Cells

Brain Cells
